# Predictors of Effectiveness of Glucagon-Like Peptide-1 Receptor Agonist Therapy in Patients with Type 2 Diabetes and Obesity

**DOI:** 10.1155/2019/1365162

**Published:** 2019-03-03

**Authors:** Alina Yu. Babenko, Daria A. Savitskaya, Yulia A. Kononova, Aleksandra Yu. Trofimova, Anna V. Simanenkova, Elena Yu. Vasilyeva, Evgeny V. Shlyakhto

**Affiliations:** Almazov National Medical Research Centre, 2 Akkuratova Street, St. Petersburg, 197341, Russia

## Abstract

**Rationale:**

It is well known that diabetes mellitus (DM) exacerbates the mechanisms underlying atherosclerosis. Currently, glucagon-like peptide-1 receptor agonists (aGLP-1) have one of the most prominent cardioprotective effects among the antidiabetic agents. However, the treatment with aGLP-1 is effective only in 50-70% of the cases. Taking into account the high cost of these medications, discovery of the predictors of optimal response to treatment is required.

**Purpose:**

To identify the predictors of the greater impact of aGLP-1 on HbA1c levels, weight reduction, and improvement in lipid profile.

**Methods:**

The study group consisted of 40 patients with type 2 DM (T2DM) and obesity who were treated with aGLP-1. The follow-up period was 24 weeks. Patients' evaluation was conducted at baseline and after 24 weeks. In addition, it included the assessment of the hormones involved in glucose and lipid metabolism and appetite regulation.

**Results:**

Patients who have initially higher BMI (body mass index), glycemia, and triglycerides (TG) had better improvement in these parameters undergoing aGLP-1 treatment. In patients with a BMI loss ≥ 5%, GLP-1 and fasting ghrelin levels were higher and ghrelin level in postnutritional status was lower. The HbA1c levels decreased more intensively in participants with higher GLP-1 levels. TG responders had lower baseline fasting glucose-dependent insulinotropic peptide (GIP) and postprandial ghrelin levels.

**Conclusion:**

The evaluation of the glycemic control, lipid profile, and GLP-1, GIP, and ghrelin levels are useable for estimating the expected efficacy of aGLP-1.

## 1. Introduction

It is well known that the rates of mortality due to cardiovascular and cerebrovascular diseases are markedly higher among people with type 2 diabetes mellitus (T2DM) [1]. Currently, the underlying mechanisms that cause T2DM and increase cardiovascular diseases (in patients with T2DM) are believed to include abnormalities in the effects of incretins and other hormones involved in glucose metabolism and food intake regulation [2].

The incretin hormones—intestinal peptide hormones, the most widely studied of which are glucagon-like peptide-1 (GLP-1) and glucose-dependent insulinotropic polypeptide (GIP)—are normally secreted in response to the oral ingestion of nutrients [2]. GLP-1 has a number of functions: augmenting insulin's response to glucose, slowing gastric emptying, and suppressing the secretion of glucagon. The latter activates the secretion of hepatic glucose and increases satiety.

GLP-1 receptor agonists (aGLP-1) have more prominent cardioprotective effects among the incretin-based antidiabetic agents. aGLP-1 demonstrate an ability to improve the prognosis for cardiovascular diseases by means of a decrease of atherosclerotic events [3]. They improve the prognosis in diabetic patients with myocardial infarction [4, 5] and may reduce arrhythmic burden and hospital admissions for heart failure worsening in diabetic patients [6].

A number of clinical studies show that aGLP-1 therapy results in a glycosylated hemoglobin (HbA1c) level reduction from 0.9 to 1.6% [7–12] and a body weight loss ranging from 0.2 to 7.2 kg [13]. The effective (target) decline of HbA1c and body weight is not observed in all patients. The group of patients with good response does not exceed 50-60% averagely [12]. Taking into account the high cost of these medications, the identification of treatment response predictors is required.

According to the literature data, the initial high level of glycemia is considered to be one of the most significant predictors of a glucose-lowering effect of aGLP-1 [14].

In addition to the influence on carbohydrate metabolism and body weight, there is an aGLP-1 effect on other cardiovascular risk factors, particularly on blood pressure (BP) [15].

According to our previous research, there was also a more significant downturn in BP in patients receiving aGLP-1 with higher degrees of hypertension, while there was no correlation between weight loss and the decrease in BP [16]. These data have shown that aGLP-1 effects on BP regulation are independent from the weight loss mechanisms.

Moreover, aGLP-1 therapy leads to favorable changes in the lipid profile and other atherogenic risk factors [17–20]. aGLP-1 therapy positively modulates inflammation in atherosclerosis in diabetic patients. [21]. The dose-dependent decline in the levels of high-sensitivity C-reactive protein (hs-CRP), plasminogen activator inhibitor 1, B-type natriuretic peptide, and endothelin-1 was demonstrated in several studies [18–20]. It is important to note that the 65% reduction in hs-CRP levels was independent on the dynamics of body and fat mass [20]. These results imply that the anti-inflammatory and potential antiatherosclerotic effects of aGLP-1 are not always associated with weight loss effects [20].

The role of the hormones involved in glucose and lipid metabolism as regard to predicting the aGLP-1 therapy efficacy is not currently clarified. Incretins, ghrelin, leptin, and adiponectin are related to this group of hormones. Consequently, the study of these hormones is also of great interest.

In obese patients, the ghrelin level in fasting plasma is lower, but the reduction in its level after partaking food is not sufficient, in comparison to adults with normal body mass index (BMI) [22]. It is probably due to an adaptive reaction in response to a positive energy balance. The administration of GLP-1 or aGLP-1 leads to ghrelin level reduction [23–25] through the neuronal mechanisms involving the hypothalamus and peripheral nervous system [25, 26]. In contrast, there is a positive correlation between the adipose tissue mass and the level of fasting plasma leptin and free leptin index. However, the negative correlation between the soluble leptin receptor and body weight was demonstrated [27]. Recent studies have shown that GLP-1 partially inhibits appetite through the regulation of the amount of soluble leptin receptors [28]. Thus, the introduction of aGLP-1 inhibits growth in soluble leptin receptors induced by weight loss, thereby maintaining the level of free leptin and preventing weight gain [27]. One year liraglutide administration to patients who previously have lost more than 12% of body weight through the hypocaloric diet triggers greater weight loss, compared to that in the control group [27]. It may be due to the reduction of leptin resistance. However, the predictive role of orexigenic and anorexigenic hormone levels in treatment efficacy has not been established so far. A lower adiponectin level has been observed in obese and diabetic populations, suggesting a reverse correlation between the adiponectin level and insulin sensitivity [29]. It has been demonstrated that aGLP-1 increase adiponectin expression and secretion.

Thus, aGLP-1 effects on hormones involved in glucose and lipid metabolism and food intake regulation are explored to a certain extent. By contrast, the association between the hormone levels and effectiveness of aGLP-1 treatment seems to be underinvestigated.

Another potential factor influencing the efficacy of aGLP-1 treatment is food behavior type. Data from Dutch researchers show that patients with an emotional type of eating behavior are less sensitive to the central effects of exenatide [30]. In a study with obese patients with T2DM who received aGLP-1 during 2 years, body mass reduction depended on the eating behavior group. The external eating behavior (*n* = 17) resulted in the smallest weight loss and the restrained eating behavior resulted in the greatest one. Meanwhile, participants with emotional and indifferent eating behavior models showed average results [31]. According to our earlier results, patients with a restrictive type of eating behavior had a tendency to a greater decline in body weight in comparison to patients with combinations of two or three behavior types [32].

Thus, aGLP-1 provide the reduction of the following cardiovascular risk factors: weight excess, hyperglycemia, hypertension, and atherogenic lipid level. The dynamics of these factors can be determined by certain predictors, and identifying them is a crucial task. The aim of our study was to identify predictors of the response to aGLP-1 therapy with regard to the reduction of blood glucose level, weight, and effect on other metabolic parameters in patients with T2DM and obesity.

## 2. Methods

### 2.1. Participants

Initially, 44 patients who met the following inclusion criteria were recruited: (i) men and women at the ages of 18 to 75 years old, with an established diagnosis of T2DM, and who signed a patient informed consent form; (ii) the presence of obesity with BMI 30 kg/m^2^ or more; and (iii) treatment with the following combinations of sugar-lowering agents: (a) biguanides, (b) biguanides + sulfonylurea medications, (c) biguanides + insulin, and (d) stable doses of glucose-lowering agents and/or hypolipidemic and antihypertensive drugs for a minimum of 3 months before inclusion.

Exclusion criteria include patients with the following conditions: (i) uncompensated hypothyroidism and/or endogenous hypercorticism; (ii) severe diseases of the cardiovascular system (congestive heart failure III-IV f. cl., uncontrolled hypertension, myocardial infarction, or acute cardiovascular event during the last 6 months); (iii) severe hepatic impairment and/or severe chronic kidney disease (C3a-C5); (iv) mental illness (including bulimia); (v) acute infectious diseases; (vi) exacerbation of chronic diseases; (vii) steroid therapy and (viii) any of the six aGLP-1 therapy contraindications to the treatment with aGLP-1 including (a) hypersensitivity, (b) diabetes mellitus type 1, (c) diabetic ketoacidosis, (d) pancreatitis in anamnesis, (e) creatinine clearance < 30 ml/min, and (f) severe gastrointestinal diseases with gastroparesis.

13 men and 31 women were recruited in the study. All participants were prescribed the following aGLP-1 treatments: 14 patients with exenatide and 30 patients with liraglutide. The follow-up period was 24 weeks. 4 patients prematurely stopped participating in the study (one patient due to acute pancreatitis development and three patients due to the lack of finances to purchase the aGLP-1 medications). The final analysis included 40 patients: 12 men (30%) and 28 women (70%); 12 on exenatide (30%) and 28 on liraglutide (70%).

87.5% of the patients were taking antihypertensive therapy, and 57.5% of the patients were taking hypolipidemic therapy. The proportions of drug groups are as follows: *β*-blockers—37.5%, sartans—45%, angiotensin-converting enzyme inhibitors—35%, diuretics—50%, calcium channel blockers—27.5%, imidozolin receptor agonists—10%, statins—57.5%, fibrates—2.5%, and omega-3 polyunsaturated fatty acids—2.5%. During the follow-up period there were no changes in therapy.

### 2.2. Study Design

#### 2.2.1. Visits Included in the Study

1. A screening visit involved the following activities:

- Elimination of endogenous hypercorticism, uncompensated hypothyroidism, and other secondary causes of obesity
- Compliance with the inclusion criteria check; individuals who met eligibility criteria were enrolled in the study and signed an informed consent
- Giving the instructions to standardize antidiabetic, lipid-lowering, and antihypertensive therapy

(2) Visit 1 (at baseline) was conducted at 3 months after therapy standardization; patients started receiving treatment with aGLP-1 (12 patients with exenatide and 28 patients with liraglutide) according to the standard regimen
(3) Visit 2 was a follow-up conducted 24 weeks after treatment initiation. Parameters estimated at all study visits are listed in Table 1

#### 2.2.2. Control Group

There are no reference ranges for GLP-1 and GIP levels. Thus, we compared GLP-1 and GIP levels in the study sample with those of the group of healthy blood donors. The control group included 19 age- and gender-matched healthy blood donors (5 men (26.3%), 14 women (73.7%), without obesity and diabetes mellitus: BMI between 22 and 25 kg/m^2^, fasting glucose level < 6.0 mmol/l, with a mean age of 48.6 years (from 26 to 65)).

#### 2.2.3. Assessments

All patients underwent an initial assessment which included a survey (complaints, medical history), physical examination with the measurement of anthropometric parameters (height, body weight, BMI calculation, and waist circumference (WC)) and vital signs (BP, heart rate (HR), and respiratory rate (RR)), and completion of the questionnaire. The BMI calculation was carried out according to the formula body mass (kg)/(height (m))^2^. Measurement of BP and HR was conducted after 5 minutes of rest, with patients seated. BP was measured 2 times with an interval of 5 minutes, and average level was recorded.

Patient's attitude to the food was assessed using two questionnaires:

1. To assess the nature of nutrition, the Dutch questionnaire of food behavior DEBQ (Dutch Eating Behavior Questionnaire) was used
2. Ratings of hunger, fullness, desire to eat, and perspective consumption were assessed using a 100 mm visual analogue scale at the fasting state (state visual analog scale to register the sensations of appetite—VAS fasting state)

The following laboratory methods were used in the study: the lipid profile was measured by the enzyme method (Roche, Germany); the level of HbA1c was detected by the method of affinity chromatography (Bio-Rad, USA); insulin level was determined using reagents and the COBAS INTEGRA 400 plus analyzer from Roche, France; levels of the hormones involved in appetite regulation and carbohydrate metabolism were evaluated using enzyme immunoassay methods for ghrelin (RayBiotech Test System (USA), leptin and GLP-1 (ARCHITECT i1000SR analyzer from Abbott (USA)), adiponectin (BioVendor), GIP (ELISA Kit, Cloud-Clone Corp.), and C-peptide (Elecsys 2010 Analyzer).

The evaluation of the degree of insulin resistance with the determination of the HOMA index of insulin resistance (НОМА-IR) was calculated according to the following formula: fasting glucose (mmol/l) × fasting insulin (mcIU/ml)/22.5. To estimate the functional reserve of *β*-cells we used an index HOMA-*β* that is calculated by the following formula: 20 × fasting insulin (mcIU/ml)/(fasting glucose (mmol/l) − 3.5). For *β*-cell function assessment, we used the meal tolerance test based on the initial level and C-peptide level determined 2 hours after the ingestion of a standard breakfast. The nutrient solution for the breakfast consisted of 190 ml of pure water and 80 g of the Clinutren Optimum mixture for enteral nutrition.

#### 2.2.4. Characteristics of the Study Group at Baseline

40 patients were finally included in the study, including 12 men (30%) and 28 women (70%), with an average age of 57.1 years (from 27 to 75) and mean diabetes duration of 11 ± 7.2 years.

All indicators were assessed at baseline and after 24 weeks of treatment.  Table 2 shows the clinical characteristics at baseline. BMI, WC, НbA1c, and glucose fasting levels were higher than normal ranges and corresponded with the criteria of diabetes and obesity. HOMA-IR exceeded the normal range more than two-fold. C-peptide level was normal in all patients and the mean value was in the middle of the normal range. After breakfast (2 h) in the meal tolerance test, an adequate growth in the C-peptide level (>50%) was achieved only in 66.7% of the participants. Most of the lipid profile components also differed from the target level for patients with diabetes. The mean total cholesterol (TC) and low-density lipoprotein (LDL) levels, the median for TG was higher and the median for high-density lipoprotein (HDL) among women was lower. However, the median for HDL among men was within target ranges. Mean systolic blood pressure (SBP) and diastolic blood pressure (DBP) levels were lower than the upper limit of normal; 42.5% of the participants had SBP > 140 mm Hg or/and DBP > 85 mm Hg.

Initial hormonal status is presented in Table 3. The initial adiponectin levels for both men and women were within the reference range, but they were less than the mean level of adiponectin (20.1 mcg/l) among healthy nonobese people in Saint Petersburg [33]. The initial leptin level for both genders was higher than the normal ranges. Fasting ghrelin level at baseline significantly differed from the reference ranges for healthy people: the mean value was more than four-fold below the lower limit of normal. After breakfast (2 h) in the meal tolerance test, an adequate downturn in ghrelin level (decrease by 30-55%) was achieved only in 40% of the participants. GLP-1 and GIP levels were compared with the control group, because there are no reference ranges for these hormones. A significant difference was observed in GLP-1 and GIP levels: in the control group these hormones were higher (Table 3(b)).

### 2.3. Statistical Analysis

Statistical analysis was performed using the program IBM SPSS Statistics 23 (Statistical Package for Social Sciences) and Statistica V. 7.0. Data are presented as numbers and percentage. Variables with a normal distribution are presented as mean ± SD and otherwise as median and interquartile range. The nonparametric Wilcoxon criterion was used to estimate the differences between dependent samples. With the help of the Mann–Whitney rank criterion, the reliability of the differences of independent variables was evaluated. The analysis of the relationship (correlation) of two quantitative traits was carried out by Spearman's test. The comparison between the different groups was carried out using a single-factor dispersion analysis (ANOVA) using a posteriori Student-Newman-Keuls criterion. For comparison of nominal variables, we used the Chi-squared test (Fisher's exact test). The differences were considered significant at *P* < 0.050.

Data in the tables are presented as mean ± standard deviation or median (25 percentile; 75 percentile).

### 2.4. Ethical Review

The research was approved by the Ethics Committee of the Almazov National Medical Research Centre (record no. 63, 14.04.14).

## 3. Results

We compared the mean reduction of BMI and HbA1c in the exenatide and liraglutide groups separately, but these parameters did not differ statistically significantly before and after treatment (Table 4).

Therefore, the results obtained in these groups were summarized.

### 3.1. What Has Changed after Treatment with aGLP-1?

Table 5 shows the clinical characteristics at baseline and after 24 weeks of treatment with aGLP-1. By the end of the therapy period (24 weeks), BMI fell by 2.4 kg/m^2^ (*P* < 0.001) and waist circumference by 6.6 cm (*P* < 0.001). Also, an improvement in carbohydrate metabolism parameters is seen: HbA1c decreases by 1.0% (*P* < 0.001) and fasting plasma glucose by 2.0 mmol/l, (*P* = 0.004).

To reveal the predictors of effective body weight loss and HbA1c reduction, we have divided the study sample into the following two subgroups: responders to the aGLP-1 treatment and nonresponders. Only body weight loss ≥ 5% from the initial level and reduction ≥ 1% in HbA1c have been considered as effective. Those subjects who showed these results were accepted as responders.

**Figure 1:**
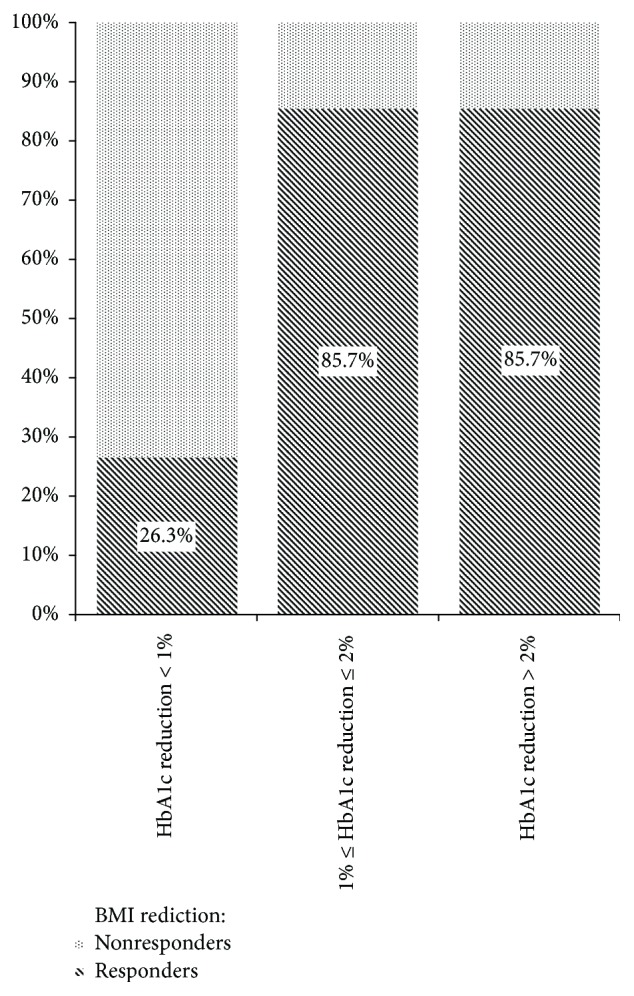
Percent of target BMI loss among groups with different HbA1c reduction levels.

An effective body weight loss (≥5% from initial) was observed in 51.5% of the participants, and target HbA1c reduction (≥1%) was observed in 39.4% of the patients. Good treatment response as BMI reduction did not always coincide with target HbA1c reduction: among patients with effective BMI loss HbA1c decline < 1% was observed in 29.4%, and among participants with effective HbA1c reduction 7.7% were nonresponders in BMI loss. But in most cases, BMI and HbA1c reduction concurred: in the group with HbA1c reduction < 1%, only 26.3% of patients had a target BMI loss; in the group with HbA1c reduction between 1% and 2% and in the group with HbA1c reduction more than 2%, 85.7% of patients subsequently had a target BMI loss (Figure 1).

#### 3.1.1. Blood Pressure and Lipid Profile

Among the characteristics of the cardiovascular system, SBP fell by 9.1 mm Hg (*P* = 0.001) and DBP by 5.8 mm Hg (*P* = 0.001). Lipid profile dynamic analysis revealed no significant changes. Similarly, there were no significant changes in C-peptide, HOMA-IR, and НОМА-*β* levels. The results are also presented in Table 5.

#### 3.1.2. Hormone Levels

A comparison of the adiponectin, leptin, ghrelin, GIP, and GLP-1 levels before and after treatment was done. Data show a significant difference only in fasting GIP level and ghrelin after meal tolerance test level: GIP and ghrelin levels were higher after treatment (Table 6).

We have also conducted the assessment of hormone level changes depending on BMI and HbA1c reduction level. The results suggest that a greater elevation of the GIP level is observed in the “nonresponders” group (Tables 7 and 8).

#### 3.1.3. The Results of the Completion of the Questionnaires

**Figure 2:**
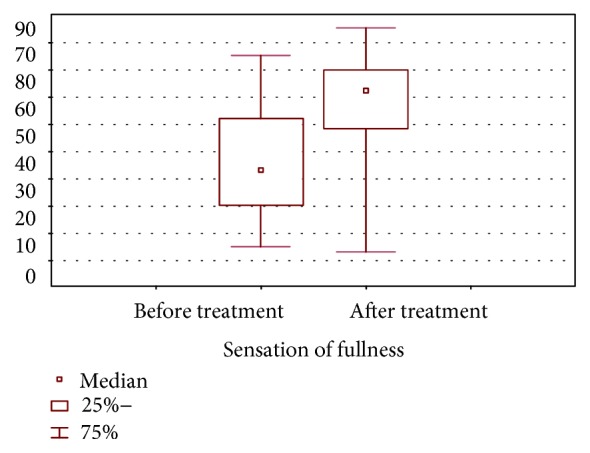
The sensations of fullness before and after treatment (VAS fasting state).

The results of the completion of the visual analogue scale to reveal attitude to the food before and after treatment display the significant rise in fullness sensation: 33.0 (17.5; 53.5) mm at baseline and 62.0 (41.5; 70.0) mm after 24 weeks of treatment (Figure 2).

The hunger, desire to eat, and perspective consumption senses were lower after treatment, but the decrease was not significant.

### 3.2. Predictors of Treatment with aGLP-1 Response

#### 3.2.1. Body Weight Loss Predictors

An effective body weight reduction (≥5% from initial) was observed in 51.5% of the participants.

Statistical analysis revealed no significant association of sex, age, diabetes duration and initial BMI with body weight loss rate.

**Figure 3:**
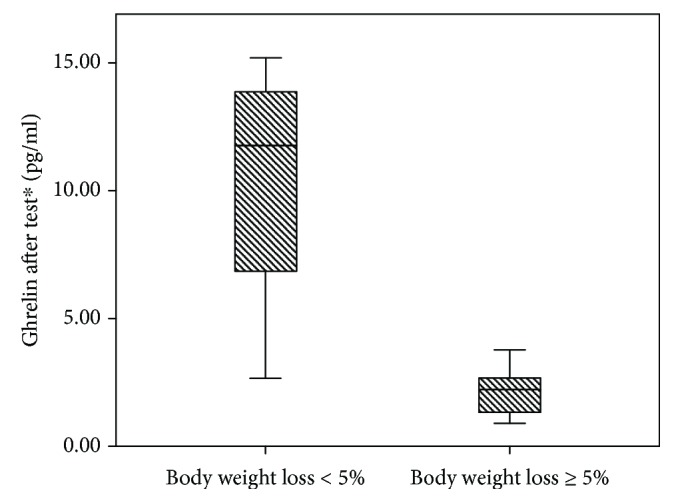
Ghrelin level after 2 hours in the meal tolerance test depending on body weight loss percentage.

The comparison of hormone (involved in appetite regulation) levels with BMI reduction rate showed that ghrelin level 2 hours after breakfast in the meal tolerance test was lower in patients with body weight reduction ≥ 5%: 2.2 (1.3; 2.7) vs. 11.8 (4.8; 14.5), *P* = 0.011 (Figure 3):

In addition, in patients with body weight loss ≥ 5% fasting ghrelin and GLP-1 levels were higher (ghrelin 1.9 (1.5; 6.1) vs. 1.4 (0.3; 2.0) pg/ml; GLP-1 0.11 (0.02; 0.14) vs. 0.01 (0.01; 0.11) mmol/l), but the differences were not significant (*P* = 0.061 and 0.242, respectively). In Pearson rank correlation analysis with the percentage of BMI loss, the major correlation was with GLP-1 level, but there was also only weak correlation with a nonsignificant *P* value: *r* = 0.384 (*P* = 0.064). Percentage of BMI loss was calculated as (BMI_baseline_–BMI_after 24 months_)/BMI_baseline_ × 100%.

Then, comparison of body weight loss rate taking into consideration appetite, hunger, fullness and type of eating behavior was performed. The only significant association was shown depending on the sensations of hunger: people with body weight loss < 5% have a stronger sense of hunger at baseline: 40.0 (25.0; 96.0) vs. 18.5 (10.0; 35.0), *P* = 0.047. The associations of body weight loss percentage with fullness, desire to eat, and perspective consumption sensations were not revealed.

The analysis of eating behavior types revealed no significant differences. Among patients with target weight loss, the percentage of those with signs of all three eating behavior types including restrained (restrained ± emotional ± external, in comparison with individuals with emotional ± external types) was higher than among patients with body weight reduction < 5%: 83.3% vs. 63.6%, but the difference was not significant (*P* = 0.229).

#### 3.2.2. HbA1c Level Reduction Predictors

**Figure 4:**
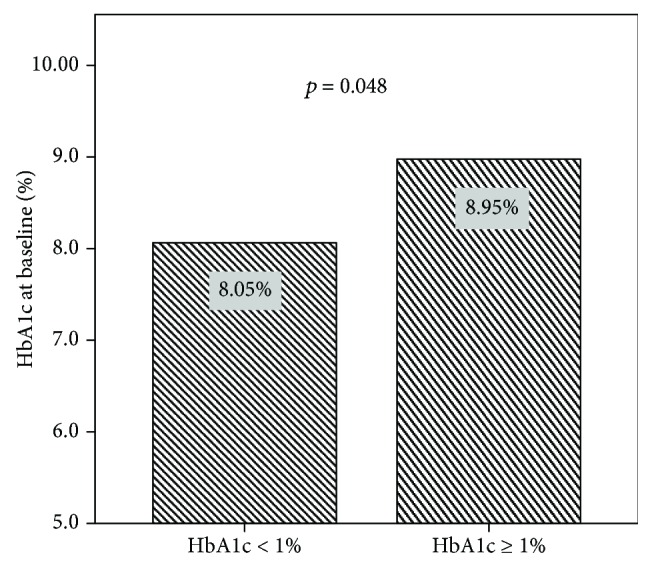
The association between the HbA1c reduction rate and HbA1c level at baseline (before aGLP-1 treatment).

**Figure 5:**
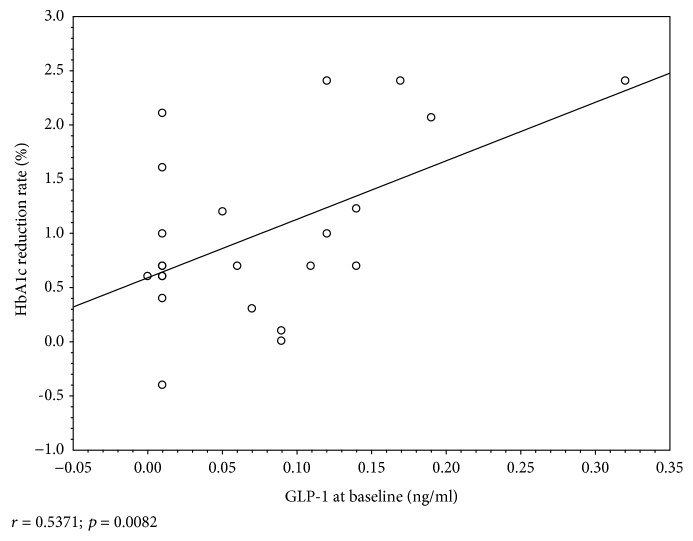
The association between the HbA1c reduction rate and GLP-1 initial level.

An effective HbA1c reduction (≥1%) was observed in 39.4% of the participants. Depending on the age, sex, and diabetes duration, the subgroups “responder” and “nonresponder” did not differ significantly. The statistical analysis showed that the HbA1c reduction rate was associated with HbA1c and GLP-1 initial levels. HbA1c reduced more intensively in patients with higher HbA1c and GLP-1 levels at baseline (Figures 4 and 5). In the Pearson rank correlation analysis, a moderate correlation was obtained between the HbA1c reduction rate and GLP-1 level with significant a *P* value: *r* = 0.537 (*P* = 0.008).

The sensations of appetite and fullness, as well as eating behavior types at baseline, were not associated with HbA1c reduction percentage.

#### 3.2.3. Lipid Profile Improvement Predictors

Table 9 shows that a more effective decline of the TG level is associated with a higher initial TG and with lower GIP and ghrelin after test levels.

TG level decrease was also more prominent in the group with target weight loss (Table 10).

## 4. Discussion

As we did not find the differences between the mean reduction of BMI and HbA1c in patients taking liraglutide and exenatide, we combined these groups.

In our study, the proportion of patients who responded well to the treatment of aGLP-1 was similar to the results of other studies [7–12]. Meanwhile, we analyzed separately the response of different metabolic parameters (weight, glycemia, and dynamics of blood pressure and lipids) to the therapy and found that it may vary according to them. So, we revealed the decline in HbA1c by more than 1% only in 39.4% of the patients who reduced their weight by 5% or more. According to the literature, 30% of the patients lost ≥5% of body weight [35]. Also, in several other studies [17] a significant improvement in lipid parameters associated with visceral obesity was noted: a decline in the TG level, an increase in the HDL level and a fall in BP. We obtained a significant decrease in BP.

Our study demonstrated that aGLP-1 is more effective in obese patients with worse glycemic control. These results coincide with the literature. The most significant weight loss and HbA1c reduction in more obese patients and in the longest diabetes duration were shown. The longer duration of diabetes was noted to be a predictor of a greater weight loss in patients on exenatide [36]. Meanwhile, unlike these researchers [36], according to our study we did not show a correlation with diabetes duration, which may be due to a higher level of C-peptide (less pronounced disturbances in the functional reserve of *β*-cells) in our study.

In other research studies, the body weight loss due to the treatment with aGLP-1 (20 mg exenatide and 1.8 mg liraglutide per day) is more successful in patients with initially higher BMI [31, 36]. Our earlier results [16] confirmed it. However, in this study, we revealed only a trend toward better weight loss in patients with a higher BMI.

We, as well as other studies, demonstrated that the dynamics of blood pressure preceded the weight reduction [15], which indicates a weight-independent mechanism for improving this parameter. In addition, in our earlier study [16], as in some other studies [15], patients with a greater severity of hypertension demonstrated a more significant decrease in blood pressure.

The change in TG level naturally depended on the dynamics of weight and reached statistical significance only in patients who reduced their weight by 5% or more. Also, the baseline level of TG was higher in those who reduced the TG level as a response to treatment by 30% or more.

There seems to be a feedback between the baseline levels and the obtained effect in almost all the studied metabolic parameters (weight, HbA1c, BP, and TG). Thus, the substrate-dependent nature of the response was noted for these metabolic parameters.

We also studied the level of hormones involved in the regulation of nutrient metabolism and appetite (GIP, GLP-1, leptin, adiponectin, and ghrelin). The basal levels of leptin and adiponectin were not shown as a predictor of the studied parameter dynamics in our research. Before treatment, we noted only a tendency to a slightly lower GIP level in weight nonresponders, but in the course of treatment the GIP level significantly increased in weight nonresponders. These differences seem logical, considering the role of GIP in the metabolism of adipose tissue (it is involved in fat intake from the gastrointestinal tract and their storage in white adipose tissue).

In our patients with T2DM and obesity, we found an abnormally low level of fasting ghrelin and no adequate reduction in the majority of the examined patients before treatment. Earlier, this fact was described by other authors in patients with obesity only and was explained as a manifestation of resistance to ghrelin. In the work of Crujeiras et al. [37], obese adults treated with a hypocaloric diet were studied. The authors noted that the low level of ghrelin before treatment and after 8 weeks of treatment was associated with an increased risk of weight regain (an increase in weight after an initial successful weight loss) in men. Our study showed that ghrelin was higher in fasting state and was lower in postnutritional status in patients with a body weight loss greater than 5%.

One more interesting finding of our study is a significant increase in ghrelin level in postnutritional status after treatment, in addition to the fact that the sense of satiety also increased significantly after 24 weeks of treatment with aGLP-1. Such dynamics of ghrelin level may indicate that patients who demonstrated good weight reduction in aGLP-1 are those who improved the sensitivity to ghrelin during the treatment. In addition, ghrelin is the regulator of GLP-1 secretion, and in the experiment, intraperitoneal injection of ghrelin to mice 15 minutes prior to oral glucose intake increased the glucose-stimulated secretion of GLP-1 and improved glucose tolerance [38]. On top of that, patients who reduced their weight by more than 5% had a higher fasting GLP-1 level. The results revealed that both the weight- and glucose-lowering effect of aGLP-1 in patients with a higher baseline level of GLP-1 indirectly confirms the results obtained in other studies about the importance of the preserved *β*-cell function for good efficacy of GLP-1 [12]. More severe GLP-1 deficiency would be expected to show the best results of aGLP-1 therapy due to the replenishment of deficiency. However, the current study showed the opposite results, which can be explained by the fact that a decrease in incretin secretion accompanies a decline in the *β*-cell reserve and even probably goes ahead of it, being a predictor of a poor response to aGLP-1 therapy. In addition, the TG responders had lower baseline fasting levels of GIP and a trend toward a lower postprandial ghrelin level, which did not reach statistical significance.

A visual analogue scale and the Dutch food questionnaire can be a useful tool for weight loss prediction. The severe hunger according to VAS was associated with a poor weight reduction on aGLP-1 therapy. Patients who had elements of the restrictive type of eating behavior often had a notable weight loss. Identifying the dominance of the emotional component in the overeating genesis is the reason for involving the psychologist in weight correction in a patient with obesity.

The limitations of our study are the low number of cases, the mixed exenatide/liraglutide group of patients, and the absence of randomization as the study was conducted within the real medical practice.

Thus, we found that in patients on aGLP-1 therapy the predictors of glucose-lowering response are HbA1c and GLP-1 initial levels and the weight loss predictors are ghrelin level 2 hours after breakfast in the meal tolerance test and a less stronger sense of hunger at baseline. Predictors of an improving lipid profile are higher initial TG and lower GIP and ghrelin after meal tolerance test levels.

## 5. Conclusion

We found that the incretin level (GLP-1 initial level) predicts the glucose-lowering response on aGLP-1 therapy, the ghrelin level 2 hours after breakfast in the meal tolerance test predicts body weight reduction, and lower GIP and ghrelin after test levels predict TG level reduction. Identification of simple and accessible predictors of the response to aGLP-1 therapy will optimize the selection of patients for this therapy and improve not only glycemic control, but also the control of metabolic parameters which are cardiovascular risk factors (BMI, waist circumference, BP, and lipids).

Identification of patients who respond well to aGLP-1 therapy on the above parameters is expected to allow outlining a group of patients who will improve their cardiovascular prognosis on aGLP-1 therapy.

## Figures and Tables

**Table 1:** Estimated parameters.

Parameters/visits	Screening	Visit 1 at baseline	Visit 2 after 24 months
Anthropometric parameters	+	+	+
Vital signs	+	+	+
Questionnaire survey		+	+
Adiponectin		+	+
GIP		+	+
Leptin		+	+
Ghrelin fasting		+	+
Ghrelin after test^∗^		+	+
GLP-1		+	+
НОМА-IR and НОМА-*β*		+	+
C-peptide fasting		+	+
C-peptide after meal test^∗^		+	+
Lipid profile		+	+
HbA1c		+	+
Review of the therapy	+	+	+

^∗^2 hours after breakfast in the meal tolerance test.

**Table 2:** Characteristics of the participants at baseline.

Characteristics	Patients on the aGLP-1, *n* = 40	Reference range
Age (years)	27-75 (57.1)^∗^	NA
Diabetes duration (years)	11 ± 7.2	NA
Body mass (kg)	114.5 ± 26.4	NA
BMI (kg/m^2^)	40.9 ± 7.6	<25
WC (cm)		
Men	125.0 (116.0; 148.0)	<90
Women	116.0 (104.8; 132.5)	<84
НbA1c (%)	8.4 ± 1.3	<6.5
Glucose fasting (mmol/l)	8.9 ± 1.8	3.3-6.1
C-peptide fasting (ng/ml)	3.5 ± 1.8	0.78-5.19
C-peptide after test^∗∗^ (ng/ml)	5.0 ± 2.8	>9.9 (↑ on >50% from baseline)
НОМА-IR	7.2 ± 4.4	<2.77
НОМА-*β* (%)	35.7 (24.5; 61.0)	≥100
TC (mmol/l)	5.2 ± 1.3	<4.5
HDL (mmol/l)		
Men	1.01 (0.79; 1.13)	>1.0
Women	1.07 (0.8; 1.3)	>1.2
LDL (mmol/l)	3.1 ± 1.1	<1.8
TG (mmol/l)	1.9 (1.5; 3.2)	<1.7
SBP (mm Hg)	138.7 ± 14.7	<140
DBP (mm Hg)	84.5 ± 8.4	<85

^∗^Min-max (mean age). ^∗∗^2 hours after breakfast in the meal tolerance test.

**Table:** (a) Hormone levels in the study group at baseline: comparison with reference range

Hormones	Patients on the aGLP-1, *n* = 32	Reference range
Adiponectin (mcg/ml)		
Men	8.9 (6.8; 17.1)	>6
Women	10.1 (8.2; 13.8)	9-12
Leptin (ng/ml)		
Men	45.0 (8.0; 79.0)	0.5-13.8
Women	32.5 (13.5; 52.5)	1.1-27.6
Ghrelin fasting (pg/ml)	1.6 (0.8; 2.7)	8.502-16.6
Ghrelin after test^∗^ (pg/ml)	2.7 (1.4; 3.5)	↓ 25-55% from baseline^∗∗^

^∗^2 hours after breakfast in the meal tolerance test. ^∗∗^See reference [34].

**Table:** (b) Hormone levels in the study group at baseline: comparison with control group

Hormones	Patients on aGLP-1, *n* = 32	Control group, *n* = 19
GLP-1 fasting (mmol/l)	0.07 (0.01; 0.14)	0.17 (0.12; 0.27)^∗^
GIP (pg/ml)	391.6 (326.6; 461.4)	574 (520.4; 744.5)^∗∗^

^∗^ *P* = 0.003. ^∗∗^*P* = 0.0001.

**Table 4:** Dynamics of body mass index and glycosylated haemoglobin in exenatide and liraglutide groups.

Therapy	BMI at baseline	BMI after 24 weeks	BMI reduction	Mean reduction of initial (%)	HbA1c at baseline	HbA1c after 24 weeks	HbA1c reduction	Mean reduction of initial (%)
Exenatide, *n* = 12	42.8	40.6	2.2	5.4	8.8	7.8	1.0	1.2
Liraglutide, *n* = 28	40.1	37.6	2.5	4.0	8.3	7.2	1.1	0.9
*P*-level				0.256				0.403

**Table 5:** Clinical characteristics at baseline and change after 24 weeks.

Characteristics	At baseline, *n* = 40	After 24 weeks, *n* = 40	*P* value
Body mass (kg)	114.5 ± 26.4	108.1 ± 25.1	<0.001
BMI (kg/m^2^)	40.9 ± 7.6	38.5 ± 7.1	<0.001
WC (cm)	122.3 ± 17.0	115.7 ± 16.2	<0.001
НbA1c (%)	8.4 ± 1.3	7.4 ± 1.3	<0.001
Glucose fasting (mmol/l)	8.9 ± 1.8	6.9 ± 1.5	0.004
SBP (mm Hg)	138.7 ± 14.7	129.6 ± 7.9	0.001
DBP (mm Hg)	84.5 ± 8.4	78.7 ± 4.9	0.001
TC (mmol/l)	5.2 ± 1.3	4.8 ± 1.1	0.215
HDL (mmol/l)	1.0 (0.8; 1.3)	1.1 (0.9; 1.3)	0.565
LDL (mmol/l)	3.1 ± 1.1	2.5 ± 1.2	0.189
TG (mmol/l)	1.9 (1.5; 3.2)	1.7 (1.4; 3.1)	0.076
C-peptide fasting (ng/ml)	3.5 ± 1.8	3.3 ± 2.1	0.569
C-peptide after test^∗^ (ng/ml)	5.0 ± 2.8	5.4 ± 2.9	0.066
НОМА-IR	7.2 ± 4.4	4.7 ± 2.7	0.089
НОМА-*β*	35.7 (24.5; 61.0)	35.3 (27.3; 57.3)	0.716

^∗^2 hours after breakfast in meal tolerance test.

**Table 6:** Hormones involved in appetite regulation levels at baseline and change after 24 weeks.

Hormone	At baseline	After 24 weeks	*P* value
GIP (pg/ml)	391.6 (326.6; 461.4)	429.9 (367.1; 504.0)	0.036
Ghrelin after test^∗^ (pg/ml)	2.7 (1.4; 3.5)	13.2 (2.1; 24.6)	0.041

^∗^2 hours after breakfast in meal tolerance test.

**Table 7:** Glucose-dependent insulinotropic polypeptide level changes after 24 weeks of treatment in different groups depending on BMI reduction.

BMI reduction	At baseline	After 24 months	*P* value
Responders	418.7 (369.2; 488.8)	431.8 (392.4; 499.3)	0.289
Nonresponders	363.0 (241.5; 419.6)	412.1 (296.2; 478.3)	0.039

**Table 8:** Glucose-dependent insulinotropic polypeptide level after 24 weeks of treatment in different groups depending on HbA1c reduction.

HbA1c reduction	At baseline	After 24 months	*P* value
Responders	396.9 (358.7; 488.8)	429.0 (396.7; 469.3)	0.375
Nonresponders	376.0 (250.2; 436.7)	430.0 (338.1; 503.1)	0.039

**Table 9:** Triglyceride level decrease predictors.

Factor (levels at baseline)	TG reduction ≥ 30%	TG reduction < 30%	*P* value
TG (mmol/l)	2.6 (2.2; 7.1)	1.6 (1.4; 2.0)	0.002
GIP (pg/ml)	331.5 (217.8; 368.5)	417.3 (380.3; 461.4)	0.019
Ghrelin after test^∗^ (pg/ml)	1.33 (0.88; 1.67)	2.65 (2.42; 11.79)	0.036

^∗^2 hours after breakfast in the meal tolerance test.

**Table 10:** Triglyceride level decrease depending on body weight loss percentage.

TG (mmol/l)	At baseline	After 24 weeks	*P* value
Body weight loss ≥ 5%	1.97 (1.51; 3.53)	1.65 (1.23; 2.62)	0.02
Body weight loss < 5%	1.95 (1.43; 2.95)	1.8 (1.47; 2.68)	0.81

## Data Availability

The data used to support the findings of this study are available from the corresponding author upon request.

## Abbreviations

aGLP-1: Glucagon-like peptide-1 receptor agonists
BMI: Body mass index
DBP: Diastolic blood pressure
SBP: Systolic blood pressure
GIP: Glucose-dependent insulinotropic polypeptide
GLP-1: Glucagon-like peptide type 1
HbA1c: Glycated hemoglobin
HDL: High-density lipoprotein
НОМА-IR: Insulin resistance index
НОМА-*β*: Index to estimate the functional reserve of *β*-cells
hs-CRP: High-sensitivity C-reactive protein
LDL: Low-density lipoprotein
T2DM: Diabetes mellitus type 2
TC: Total cholesterol
TG: Triglycerides
WC: Waist circumference.
